# Leptin Level and Skipping Breakfast: The National Health and Nutrition Examination Survey III (NHANES III)

**DOI:** 10.3390/nu8030115

**Published:** 2016-02-25

**Authors:** Keiko Asao, Amandine Sambira Marekani, Jessica VanCleave, Amy E. Rothberg

**Affiliations:** 1 Department of Preventive Medicine, University of Tennessee Health Science Center, 66 N. Pauline Street, Ste. 633, Memphis, TN 38105, USA; asambir@hotmail.com; 2 Church Health Center Wellness, 1115 Union Avenue, Memphis, TN 38104, USA; vancleavej@churchhealthcenter.org; 3 Division of Metabolism, Endocrinology and Diabetes, Department of Internal Medicine, University of Michigan, Domino’s Farms Lobby G, 24 Frank Lloyd Wright Drive, Ann Arbor, MI 48105, USA; arothber@med.umich.edu; 4 Department of Nutritional Sciences, University of Michigan, School of Public Health, 1415 Washington Heights, Ann Arbor, MI 48109, USA

**Keywords:** breakfast, leptin, the National Health Examination and Nutrition Survey (NHANES)

## Abstract

Skipping breakfast is a common dietary habit considered to be unhealthy. However, the mechanisms underlying skipping breakfast have not been fully explored. Leptin is a hormone that regulates food intake and energy storage and secretes in a diurnal rhythm with lowest levels in the morning. We examined the association between the serum leptin level and skipping breakfast in 5714 adults in the U.S. National Health and Nutrition Examination Survey III, 1988–1994. We defined breakfast as any food or beverage consumed between 5:00 a.m. and 10:00 a.m. using a single 24-h recall. Skipped breakfast was seen in 13.1%. In the logistic regression models with and without adjusting for adiposity and sex, leptin levels were not associated with skipping breakfast. After adjusting for age, race/ethnicity, and time of venipuncture, the association remained insignificant. After further adjusting for potential confounders: physical activity, alcohol intake, smoking and diabetes and after further adjusting for: dietary factors, insulin and glucose levels, there was a 9% and 11%–12%, respectively, statistically significantly higher likelihood of skipping breakfast if the leptin level was more than 50% greater. Further investigation into the biological reasons for skipping breakfast may be useful for promoting healthy lifestyles.

## 1. Introduction

Skipping breakfast is thought to be an unhealthy dietary habit, although a recent study suggested that evidence to support this position is wholly lacking [1]. Based on a national survey, approximately one quarter of American adults skip breakfast [2,3]. About half of young adults report that they are “too rushed in the morning to eat a healthy breakfast”. Various sociodemographic and lifestyle factors have been reported as correlates to breakfast skipping: younger age [4], poverty [4], African American race [4], male sex [5], lower education [4], non-Southern regions of the United States of America [4], urban residence [4], smoking [5], alcohol use [5], and infrequent exercise [5]. Apart from these sociodemographic and lifestyle factors, anecdotally, some individuals report that they “do not feel hungry” in the morning or self-identify as “not a morning person”, and, therefore, defer breakfast.

Energy homeostasis is tightly regulated. Leptin is a hormone that is secreted primarily by white adipose tissue and circulates at levels positively correlated with fat mass, thus reflecting the amount of stored energy [6]. Leptin levels also change with acute changes in energy availability. Fasting decreases circulating leptin levels, and similarly, a low level of leptin is correlated with energy restriction [7]. Leptin is known to be secreted in a diurnal rhythm with lower levels in the morning and higher levels at night [8].

We hypothesize that people do not eat breakfast regularly because they have a relatively higher leptin level in the morning.

To our knowledge, there is limited information on the association between fasting leptin level and skipping breakfast [9]. A previous study that measured leptin was limited due to its sample size (34 subjects) and lack of adjustment for potential confounders [9]. Therefore, we examined the association between the fasting serum leptin levels and skipping breakfast in the National Health and Nutrition Examination Survey (NHANES) III, a nationally representative cross-sectional study of the U.S. population.

## 2. Methods

Study subjects: NHANES III was conducted using a multistage sampling design representative of the non-institutionalized civilian population in the United States from 1988 to 1994 [10]. Serum leptin levels were measured in 6415 adults and available in the NHANES III public dataset [11]. The serum leptin levels were measured in the surplus sera for those who were 20 years old or older and who were randomly assigned to the morning examination after overnight fasting [11]. NHANES III collected blood samples at a greater volume than usually necessary for out-of-range results that require repeated assays [11]. In addition, for the present analysis, pregnant or lactating women were excluded (*n* = 147). Then, participants whose dietary recall was judged other than “reliable and complete” were excluded (*n* = 173). Participants who had missing bioelectrical impedance measurements, a negative calculated percentage body fat (%BF), or calculated total body water exceeding 80% of their body weight [12] (*n* = 376) were omitted. Finally, participants whose phlebotomy was done past 1:00 p.m. (*n* = 5) were removed. In total, 5714 adults were included in the analysis.

Variables of interest: Dietary intake was assessed using a computerized, single 24-h recall for the previous day of the examination, including the timing of each meal. We defined breakfast as any food or beverage consumed between the hours of 5:00 a.m. and 10:00 a.m. Because dietary intake was assessed using a single 24-h recall, breakfast eating assessed in this study is not necessarily habitual. During the dietary recall interview, participants were not asked to report plain drinking water [13]. Serum leptin assays were performed by Linco Research, Inc. (St. Louis, MO, USA) using a radioimmunoassay [11].

Covariate assessment: %BF was considered to be the major covariate in the association since leptin levels are positively correlated with %BF and the assumption that obese individuals tend to skip breakfast [2,4]. We also conducted supplemental analysis using the sum of three skinfold thicknesses in the place of %BF.

%BF was estimated using the bioelectrical impedance method (Valhalla Scientific Body Composition Analyzer 1990 B). Bioelectrical impedance was not obtained for pregnant women (who were excluded in this analysis) and individuals with cardiac pacemakers, limb amputations, or metal pins in their hips. We used the prediction formula from the literature [12] to derive the fat-free mass, which was subtracted from body weight to yield total body fat. %BF was then defined as the percentage of total body fat divided by weight. Skinfold thickness was measured using a Holtain skinfold caliper according to a standardized protocol [14]. The sum of the skinfold thickness of triceps, subscapular, and suprailiac measurements was derived and then transformed using logarithms [15,16]. Skinfolds too large for the caliper were treated as 50 mm. Height and weight were measured according to a standardized protocol [14], and body-mass index was calculated as body weight in kilograms divided by the square of height in meters. We then categorized %BF and the sum of three skinfold thicknesses into sex-specific quartiles.

Several other factors are known to be potentially associated both with leptin levels and with skipping breakfast. We attempted to adjust for these factors in our analysis. Information on these covariates was collected in standardized interviews [11,17]. Race/ethnicity was categorized into four groups: non-Hispanic white, non-Hispanic black, Mexican American, and others. The level of physical activity was classified into four categories (vigorously active, moderately active, lightly active, and sedentary) based on the frequency and intensity of reported physical activities [18]. We defined “vigorously active” as having ≥3 times a week of physical activity with a metabolic equivalent level ≥6 for individuals ≥60 years old and ≥7 for individuals <60 years old; “moderate” as ≥5 times a week, <3 times of which are vigorous as defined above; “light” as having at least one activity a week but not “vigorously active” or “moderately active”; and “sedentary” as having no activity. Subjects were classified as “ever smokers” if they reported that they had smoked at least 100 cigarettes during their lives; among “ever smokers”, we defined them as “current smokers” if participants responded “yes” to the question “Do you smoke cigarettes now?” Otherwise, they were classified as “never smokers”. Having diabetes was defined as a positive response to the question “Have you ever been told by a doctor that you have diabetes or sugar diabetes?” at a time other than during pregnancy.

As previously mentioned, dietary intake was assessed using a computerized, single 24-h recall for the previous day of the examination. We used the estimated dietary energy intake (total, percentage from carbohydrate, and percentage from fat) available in the public NHANES dataset based on the University of Minnesota’s Nutrition Coordinating Center. Alcohol intake was assessed using a standardized questionnaire, not from the 24-h recall. Current alcohol drinkers were classified as people who consumed at least 12 drinks in their entire life and drank at least 1 day in the past year. Among current drinkers, the number of drinks per day was derived based on the average drinks per day and the number of days when the participants drank in the past 12 months [19], and categorized into >0 and <1, ≥1 and <2, and ≥2 drinks. All non-current drinkers were categorized into 0 drinks a day. Caffeine intake was captured from the 24-h recall and was classified into 0, >0 and <100, ≥100 and <200, and ≥200 mg/day based on the dietary nutrient estimates. We defined a night eater as anyone eating anything between 10:00 p.m. and 2:00 a.m. Plasma glucose (mg/dL) was measured using an enzymatic method and serum insulin (µU/mL) was measured with a radioimmunoassay [20].

Statistical analyses: We performed all statistical analyses using SAS 9.4 software (Version 9.4, Cary, NC, USA) [21]. All analyses accounted for the complex, multistage, stratified, cluster-sampling design of NHANES III using the sample weights provided by the survey designer as part of the public data [10]. We used the SAS survey procedures with “nomcar” to handle missing values as missing at not completely random. The type I error was set at 0.05.

We first described the characteristics of the study participants using mean and standard deviation, with logarithmic transformation when appropriate, and percentage by breakfast eating status. When we transformed variables using logarithms, we presented them as geometric means [22]. We tested the difference in the distribution of the characteristics by breakfast eating status with student’s *t*-tests, one-way analysis of variance (ANOVA), and chi-square tests as appropriate.

We then constructed logistic regression models to test the association between skipping breakfast and serum leptin level. Since leptin level is known to be skewed to the right, we transformed the level using a base-10 logarithmic transformation. Due to the considerable difference in leptin level by sex and adiposity, all analyses were stratified by sex and adiposity first before combining the sex and adiposity groups when appropriate.

For each analysis, we constructed four nested models: (1) a basic model including leptin as an independent variable and breakfast eating as the dependent variable; (2) a model adjusting for age, race/ethnicity, time of venipuncture, sex when strata for sex were combined, and adiposity when strata for adiposity were combined; (3) a model with other potential confounders (physical activity, alcohol intake, smoking) in addition to the factors included in Model 2; and (4) a model further adjusting for total caloric intake, percentage caloric intake from carbohydrate and fat per day, and nighttime eating. The results were expressed as the odds ratio (OR) of skipping breakfast per one-unit change in log-transformed leptin level (base 10) (OR_logLeptin_) but interpreted as the converted odds ratio of skipping breakfast per specific percentage increment in leptin level (OR_%Leptin_). To convert OR_logLeptin_ into OR_%Leptin_ for the p percentage of the change in leptin level, we used the following formula: ${\mathrm{OR}}_{\%\mathrm{Leptin}}={{\mathrm{OR}}_{\mathrm{logLeptin}}}^{log10\left(1+p\times 0.01\right)}$.

## 3. Results

Among the 5714 eligible participants, 932 participants (13.1%, weighted) skipped breakfast. The characteristics of participants who ate *vs.* skipped breakfast are displayed in Table 1. Participants who skipped breakfast were more likely to be younger, male, non-Hispanic black or Mexican American, and a current smoker than those who did not skip breakfast. Participants who skipped breakfast consumed a higher proportion of caloric intake from carbohydrates than those who ate breakfast, but total caloric intake and percentage caloric intake from fat were similar by breakfast eating status. Participants who skipped breakfast consumed more alcohol and ate at nighttime (between 10:00 p.m. and 2:00 a.m.) more often than those who ate breakfast. The indices for adiposity were similar by breakfast eating status. Participants who skipped breakfast had a slightly later time of blood collection (~12 min) than those who ate breakfast. Insulin and glucose levels were not statistically different by breakfast eating status.

Leptin level was not significantly different by breakfast eating status either in men or in women (Table 1) when compared by *t*-test. The association was examined in detail using logistic regression models (Table 2). As described in the method section, we first examined the association stratified by sex-specific quartiles of %BF and sex. Since sex did not modify the association between leptin and skipping breakfast (*i.e.*, all interaction terms by sex were not statistically significant), we combined men and women for each sex-specific quartile of %BF. Next, since the quartiles of %BF did not modify the association between leptin and skipping breakfast (*i.e.*, all interaction terms by the quartiles of %BF were not statistically significant), we combined all quartiles of %BF.

The models combining both sex and all quartiles of %BF (the bottom row of Table 2) were constructed stepwise. Models 1 and 2, which adjusted for the quartiles of %BF and sex (Models 1 and 2), age, race/ethnicity, and time of venipuncture (Model 2), did not show a significant association between leptin level and skipping breakfast. After further adjusting for physical activity, alcohol intake, smoking, and diabetes (Model 3), the association showed statistical significance. One-unit increment in log-transformed leptin level (base 10) was associated with a 1.6 times higher likelihood of skipping breakfast (Model 3). By using the conversion formula, this finding can be interpreted as a 9% higher chance of skipping breakfast if leptin level is 50% higher. The association remained statistically significant after further adjusting for total caloric intake, percentage caloric intake from carbohydrate and fat, nighttime eating, and caffeine intake (Model 4) as well as for insulin and glucose (Model 5). After adjusting for dietary factors and insulin and glucose, a 50% higher leptin level was associated with an 11%–12% higher likelihood of skipping breakfast. We repeated the analysis using the sum of the three skinfold thicknesses instead of %BF (Appendix Table A1). The results using skinfolds were similar to the findings from the analysis using %BF.

## 4. Discussion

In our study, a higher fasting leptin level was not associated with a higher likelihood of skipping breakfast, but was associated with skipping breakfast after adjusting for potential confounders. After adjusting for %BF, sociodemographic factors, lifestyle factors, insulin, and glucose, a 50% higher leptin level was associated with a 9%–12% higher likelihood of skipping breakfast. Our study is one of the first to examine an association between a fasting leptin level and skipping breakfast. In another study that measured leptin and breakfast patterns, no such association was found [9].

It should be noted that the aforementioned significant association was observed only after adjusting for confounders beyond sex and %BF. We suspect that this is partly due to the wide range of leptin levels previously reported. For example, the median for fasting serum leptin levels in the U.S. general population were 13.2 (7.7, 21.8) and 4.4 (2.7, 7.4) (25 and 75 percentiles, respectively) for women and men, respectively [23]. This study is based on a large sample size, providing a high power for statistical tests. Based on *post hoc*, unweighted power analyses, to detect an approximately 10% higher likelihood of skipping breakfast for a 50% higher leptin level, the power for men (N = 2705) was 0.78 and for women (N = 3009) was 0.82 assuming 0.05 for type I error in this study. Therefore, this study is likely to have a relatively high power when adjusting for sex and %BF.

If the associations were significant only after further adjustment of multiple confounders, these associations can be interpreted based on various mechanisms. First, the association between leptin level and skipping breakfast can be mediated by dietary factors. Leptin is a signal for energy storage. Those with a higher leptin level in the morning may be predisposed to skipping breakfast due to decreased appetite. A change in fasting leptin level can result in a change in the overall leptin level or a shift in leptin diurnal rhythm, resulting in a higher level in the morning by delaying the physiologic decrease. Moreover, this change can be enhanced by certain dietary habits. Previous studies have shown that a change in eating patterns, *i.e.*, a change in the composition and number of meals, may change leptin level and its diurnal rhythm. For example, a diet rich in carbohydrates [24] or a high glycemic index diet [25] induced higher leptin levels at ≥4 h after breakfast. Some studies show that shifting meal times alter the leptin diurnal pattern [26]. In Model 5, we accounted for total caloric intake, percentage caloric intake from carbohydrate and fat, caffeine intake, and nighttime eating. However, these adjustments did not attenuate the association between breakfast eating and leptin. Therefore, dietary factors may not be sufficient mediators of this association.

Insulin is also considered to be a possible mediator for the association between leptin level and skipping breakfast. Increasing insulin level raises leptin level during a 72-h euglycemic-hyperinsulinemic clamp [27]. In our study, the association between breakfast eating and leptin level remained significant after adjusting for insulin level. However, the association we observed between leptin level and breakfast eating remained after adjusting for these factors. Therefore other possible explanations of the observed association may need to be explored.

Since the design of this study is cross-sectional, we were unable to assess directionality of the association. Previous evidence showed that short-term fasting decreased the leptin level [28]. Therefore, we cannot exclude the potential for reverse causality, *i.e.*, that frequently skipping breakfast increases the leptin level.

We considered multiple potential confounders for the observed associations. In our study, the association between breakfast eating and leptin was significant only after adjusting for these lifestyle factors. Albeit inconsistently, previous studies have provided information that smoking [29,30,31] and alcohol consumption [29,32] are correlated with higher leptin levels, while smoking [2,3] and alcohol consumption [29,32] are associated with skipping breakfast as shown herein. Because we observed an association between leptin and skipping breakfast after adjusting for these factors, the observed association should not solely derive from these lifestyle factors.

Eating breakfast has been recommended as part of a healthy lifestyle [33]. Studies have shown that eating breakfast is associated with a better diet quality [34], glucose tolerance profile (second meal phenomenon) [35], and task performance and mood [36]. However, it is controversial whether breakfast eating prevents weight gain or promotes weight loss [1,37]. For example, a recent randomized clinical trial demonstrated no effect on weight loss by eating breakfast or skipping breakfast [38]. Conversely, a systematic review of observational studies linked breakfast eating with a lower likelihood of obesity [39]. Benefits of eating breakfast may depend on the quality of breakfast and warrant re-evaluation for various outcome measures.

This study has a large sample size, representativeness for the U.S. general population, and extensive sets of covariates as its strengths. However, there are some limitations in this study. First, NHANES did not collect direct information on the habits of breakfast eating. We categorized breakfast eating status based on one 24-h dietary recall based on any food intake on the day of the examination. We assumed that people who missed their breakfast on the day of the dietary recall are likely to miss their breakfast habitually, but we do not have data to validate the assumption. Second, this study did not account for potential confounders that might alter the diurnal variation of leptin, such as sleep. However, previous studies showed inconsistency in the change of the leptin level by sleep restriction [40,41]. Therefore, presently, it is not clear how lack of the information on sleep may have impacted the current study. Third, this study did not have information on some hormones related to hunger and appetite, such as ghrelin [42] and peptide YY [43]. Future studies with comprehensive panels of the hormones will help to understand how the association between skipping breakfast and leptin relates to other relevant hormones.

As a conclusion, we found that people who do not eat breakfast have a higher leptin level but only after adjusting for multiple potential confounders. The mechanisms of this association appear to be independent of dietary habits and insulin. Considering the high prevalence of skipping breakfast and potential health benefits of eating breakfast, further investigation into the biological mechanisms for people’s behaviors may be useful for promoting healthy lifestyles.

## Figures and Tables

**Table 1:** Characteristics of participants by skipping breakfast, the National Health and Nutrition Examination Survey (NHANES) III, 1988–1994.

	Overall	Skip Breakfast	Eat Breakfast	*p*-Value
N	5714	932 (13.1%)	4782	-
Age, years	44.3 (0.6)	35.9 (0.6)	45.6 (0.7)	<0.01
Women (%)	48.1% (0.9%)	43.9% (3.2%)	53.1% (1.0%)	<0.01
Race/ethnicity, %	-	-	-	<0.01
Non-Hispanic Whites	77.6% (1.5%)	65.2% (2.5%)	79.5% (1.6%)	-
Non-Hispanic Blacks	9.8% (0.7%)	19.7% (1.6%)	8.4% (0.6%)	-
Mexican Americans	4.7% (0.5%)	7.0% (0.8%)	4.4% (0.5%)	-
Others	7.8% (1.1%)	8.1% (2.0%)	7.7% (1.1%)	-
Physical activity (%)	-	-	-	0.69
Vigorous	6.9% (0.5%)	8.3% (1.6%)	6.6% (0.6%)	-
Moderate	36.7% (1.4%)	34.8% (2.5%)	36.9% (1.6%)	-
Light	43.4% (1.1%)	43.0% (2.9%)	43.4% (1.2%)	-
Sedentary	13.1% (1.0%)	13.8% (1.9%)	13.0% (1.0%)	-
Smoking cigarettes (%)	-	-	-	<0.01
Never	45.9% (1.1%)	47.4% (3.3%)	45.7% (1.1%)	-
Former	27.0% (0.9%)	18.3% (2.4%)	28.3% (1.0%)	-
Current	27.1% (1.0%)	34.3% (3.0%)	26.0% (1.1%)	-
Self-reported diabetes (%)	3.5% (0.3%)	2.6% (0.8%)	3.7% (0.4%)	0.34
Caloric intake	-	-	-	-
Total, kcal/day	2236.6 (27.5)	2195.3 (61.1)	2242.9 (31.1)	0.50
% from carbohydrate	49.5 (0.4)	47.1 (0.7)	49.9 (0.4)	<0.01
% from fat	34.2 (0.3)	35.0 (0.6)	34.1 (0.3)	0.16
Current alcohol intake, drink/day	-	-	-	<0.01
None	43.9% (1.8%)	35.7% (2.6%)	45.1% (2.0%)	-
0–1	41.6% (1.4%)	43.0% (3.0%)	41.4% (1.6%)	-
1–2	8.4% (0.7%)	11.5% (2.1%)	7.9% (0.8%)	-
≥2	6.1% (0.6%)	9.8% (1.5%)	5.5% (0.7%)	-
Caffeine intake, mg/day	-	-	-	<0.01
None	9.1% (0.6%)	18.8% (1.5%)	7.6% (0.6%)	-
0–100	30.2% (1.1%)	38.9% (2.7%)	28.9% (1.2%)	-
100–200	18.4% (0.9%)	19.1% (2.4%)	18.3% (1.0%)	-
≥200	42.4% (1.2%)	23.2% (2.6%)	45.3% (1.3%)	-
Night eater (%)	17.6% (1.0%)	27.0% (2.9%)	16.2% (0.9%)	<0.01
Body mass index, kg/m^2^, %	26.5 (0.2)	26.6 (0.4)	26.5 (0.2)	0.83
Percent body fat (%)	-	-	-	-
Men	24.2 (0.3)	23.9 (0.6)	24.2 (0.3)	0.58
Women	35.2 (0.3)	35.1 (0.9)	35.2 (0.3)	0.93
Subcutaneous fat thickness, mm *	-	-	-	-
Men	49.2 (1.0)	47.9 (1.0)	49.4 (1.0)	0.49
Women	57.9 (1.0)	56.3 (1.0)	58.1 (1.0)	0.42
Time at venipuncture, hours from midnight	9.2 (0.0)	9.4 (0.0)	9.2 (0.0)	<0.01
Leptin, µg/L *	-	-	-	-
Men	4.5 (0.1)	4.3 (0.3)	4.6 (0.1)	0.52
Women	12.7 (0.4)	12.6 (0.9)	12.7 (0.4)	0.89
Glucose, mg/dL	99.8 (0.4)	98.6 (1.1)	100.0 (0.4)	0.26
Insulin, µU/mL	10.6 (0.3)	11.0 (0.5)	10.5 (0.3)	0.30

* Geometric means and S.E.M.

**Table 2:** Odds ratios for skipping breakfast by a one-unit increment in log-transformed leptin level (base 10), stratified by percent body fat, the National Health and Nutrition Examination Survey (NHANES) III, 1988–1994.

% Body Fat	Sex	Model 1	Model 2	Model 3	Model 4	Model 5
Q1	Men	0.39 (0.12, 1.24)	1.03 (0.31, 3.42)	1.21 (0.36, 4.07)	1.77 (0.43, 7.24)	1.64 (0.39, 6.93)
	Women	1.40 (0.45, 4.36)	1.54 (0.55, 4.33)	2.20 (0.76, 6.34)	3.95 (0.70, 22.37)	4.05 (0.72, 22.89)
	Overall	0.67 (0.33, 1.38)	1.29 (0.63, 2.63)	1.54 (0.84, 2.82)	2.23 (0.99, 5.01)	2.15 (0.96, 4.79)
Q2	Men	1.57 (0.60, 4.16)	**2.89 (1.03, 8.10)**	**2.85 (1.01, 8.04)**	**10.91 (2.94, 40.48)**	**11.13 (2.99, 41.51)**
	Women	1.61 (0.43, 5.96)	1.77 (0.41, 7.63)	1.42 (0.29, 7.00)	1.48 (0.26, 8.33)	1.45 (0.26, 8.24)
	Overall	1.60 (0.76, 3.39)	**2.37 (1.12, 5.02)**	**2.34 (1.08, 5.05)**	**4.05 (1.80, 9.14)**	**4.05 (1.79, 9.17)**
Q3	Men	1.16 (0.18, 7.43)	1.90 (0.33, 11.15)	2.99 (0.68, 13.17)	2.11 (0.44, 10.09)	2.10 (0.45, 9.78)
	Women	0.84 (0.13, 5.29)	0.82 (0.10, 6.64)	1.00 (0.09, 11.12)	1.18 (0.11, 12.59)	1.38 (0.13, 14.95)
	Overall	1.02 (0.24, 4.22)	1.35 (0.31, 5.78)	1.63 (0.41, 6.48)	1.62 (0.40, 6.53)	1.60 (0.40, 6.41)
Q4	Men	0.81 (0.23, 2.84)	1.20 (0.35, 4.13)	0.80 (0.18, 3.56)	0.55 (0.11, 2.83)	0.55 (0.10, 3.11)
	Women	0.46 (0.13, 1.65)	0.51 (0.12, 2.10)	0.52 (0.12, 2.23)	0.43 (0.09, 2.05)	0.36 (0.07, 1.86)
	Overall	0.60 (0.26, 1.38)	0.76 (0.33, 1.76)	0.70 (0.30, 1.64)	0.58 (0.25, 1.34)	0.55 (0.23, 1.31)
Overall	-	0.87 (0.56, 1.35)	1.48 (0.98, 2.24)	**1.60 (1.03, 2.51)**	**1.88 (1.18, 2.98)**	**1.85 (1.15, 2.97)**

Q1–Q4: quartiles 1 to 4. Model 1 is a basic model including log-transformed leptin, the sex-specific quartiles of percent body fat (for sex-specific overall models and the overall model), and sex (the overall model) as independent variables. Model 2 included age, race/ethnicity, and time of venipuncture as independent variables in addition to the variables included in Model 1. Model 3 included physical activity, alcohol intake, smoking, and diabetes in addition to the variables included in Model 2. Model 4 included total caloric intake, percentage caloric intake from carbohydrate and fat per day, caffeine intake, and nighttime eating in addition to the variables included in Model 3. Model 5 included insulin and glucose in addition to the variables included in Model 4. Odds ratios in bold indicate *p*-value < 0.05.

## Abbreviations

The following abbreviations are used in this manuscript:

NHANES: National Health and Nutrition Examination Survey
%BF: percentage body fat
OR: odds ratio

## Appendix

**Table A1:** Odds ratios for skipping breakfast by a one-unit increment in log-transformed leptin level (base 10), stratified by the sum of subcutaneous fat thickness, the National Health and Nutrition Examination Survey (NHANES) III, 1988–1994.

Sum of Three Skinfold Thicknesses	Sex	Model 1	Model 2	Model 3	Model 4	Model 5
Q1	Men	1.16 (0.28, 4.88)	5.87 (0.74, 46.41)	5.52 (0.72, 42.67)	**10.12 (1.21, 84.41)**	**9.13 (1.12, 74.56)**
	Women	1.10 (0.22, 5.66)	2.16 (0.32, 14.43)	2.14 (0.34, 13.62)	2.35 (0.32, 17.34)	2.40 (0.32, 17.81)
	Overall	1.18 (0.37, 3.81)	1.34 (0.58, 3.09)	3.28 (0.79, 13.63)	3.51 (0.72, 17.05)	3.45 (0.70, 16.94)
Q2	Men	1.15 (0.27, 4.82)	2.27 (0.35, 14.66)	1.98 (0.26, 14.98)	3.03 (0.39, 23.79)	3.13 (0.41, 24.02)
	Women	0.96 (0.29, 3.19)	1.27 (0.28, 5.78)	1.04 (0.15, 7.09)	1.42 (0.22, 9.36)	1.45 (0.22, 9.61)
	Overall	1.06 (0.52, 2.15)	**1.84 (1.01, 3.37)**	1.60 (0.69, 3.70)	2.21 (0.97, 5.04)	2.24 (0.98, 5.12)
Q3	Men	1.97 (0.44, 8.78)	**4.91 (1.22, 19.69)**	4.43 (0.98, 20.03)	**4.98 (1.22, 20.33)**	**4.86 (1.20, 19.68)**
	Women	1.68 (0.23, 12.09)	5.31 (0.44, 63.57)	7.69 (0.62, 96.15)	9.65 (0.73, 126.98)	7.96 (0.60, 104.83)
	Overall	1.88 (0.60, 5.85)	3.14 (0.94, 10.45)	**4.61 (1.22, 17.34)**	**5.90 (1.70, 20.52)**	**5.82 (1.66, 20.45)**
Q4	Men	0.25 (0.05, 1.35)	0.77 (0.13, 4.39)	0.54 (0.08, 3.95)	0.29 (0.04, 1.99)	0.29 (0.04, 1.91)
	Women	0.58 (0.17, 2.07)	0.88 (0.19, 4.07)	0.96 (0.20, 4.70)	0.52 (0.10, 2.61)	0.48 (0.10, 2.40)
	Overall	0.36 (0.09, 1.36)	0.85 (0.29, 2.51)	0.73 (0.17, 3.03)	0.41 (0.10, 1.66)	0.40 (0.10, 1.62)
Overall	-	0.94 (0.53, 1.67)	**2.17 (1.13, 4.19)**	**2.00 (1.04, 3.84)**	**1.91 (1.01, 3.63)**	1.87 (0.97, 3.63)

Q1–Q4: quartiles 1 to 4. Model 1 is a basic model including log-transformed leptin, the sex-specific quartiles of S (for sex-specific overall models and the overall model), and sex (the overall model) as independent variables. Model 2 included age, race/ethnicity, and time of venipuncture as independent variables in addition to the variables included in Model 1. Model 3 included physical activity, alcohol intake, smoking, and diabetes in addition to the variables included in Model 2. Model 4 included total caloric intake, percentage caloric intake from carbohydrate and fat per day, caffeine intake, and nighttime eating in addition to the variables included in Model 3. Model 5 included insulin and glucose in addition to the variables included in Model 4. Odds ratios in bold indicate *p*-value < 0.05.
